# DNA stable isotope probing reveals hidden microbial carbon processing pathways in agricultural soils

**DOI:** 10.1128/aem.01504-25

**Published:** 2025-10-29

**Authors:** Malak M. Tfaily

**Affiliations:** 1 Department of Environmental Science, University of Arizona 8041, Tucson, Arizona, USA; Michigan State University, East Lansing, Michigan, USA

**Keywords:** DNA-SIP, tillage, carbon processing, resource partitioning, bacterial communities

## Abstract

Agricultural soil management plays a key role in driving the microbial communities responsible for soil carbon cycling, yet mechanistic understanding of these effects has been limited by the inability to directly observe microbial activity *in situ*. Traditional molecular approaches like 16S rRNA sequencing show community composition but do not distinguish between which organisms are actively processing what substrates in the field. A study by M. Schaedel, C. Koechli, and D. H. Buckley in *Applied and Environmental Microbiology* (91:e00933-25, 2025, https://doi.org/10.1128/aem.00933-25) highlights how DNA stable isotope probing overcomes these shortcomings and offers molecular evidence that long-term tillage history reorganizes bacterial carbon processing pathways, as well as the temporal dynamics of substrate assimilation and functional coherence of soil microbial communities.

## COMMENTARY

The response of soil microbial communities to management practices is among one of the most persistent challenges remaining to understanding biogeochemical processes in terrestrial ecosystems (1). Culture-independent toolkits are revolutionary in their ability to illuminate microbial diversity (2), yet they have one critical shortcoming in that they represent potential and not realized activity. 16S rRNA gene sequencing informs us of the bacteria that are present, and metagenomics that of their metabolic potential (3), but neither method can identify with certainty which organisms are actually degrading specific substrates in the field (4).

This knowledge gap is particularly concerning because it shapes our perception of how environments should or will respond to management practices. In agricultural systems, management creates complex environmental gradients that influence microbial activity in ways that cannot be predicted from taxonomic composition alone (5). The study by Schaedel et al. (6) tackles this problem directly using DNA stable isotope probing (DNA-SIP) (7, 8) to directly track bacterial carbon assimilation in real time. This approach represents a fundamental shift from inferential to observational microbial ecology, providing insights into how bacteria actually process carbon under different management regimes (9).

DNA-SIP (7–9) works by giving microorganisms substrates labeled with heavy isotopes (in this study, ¹³C-labeled xylose and cellulose). Then, these labels get tracked into the bacteria’s DNA through density gradient centrifugation. This technique has several crucial advantages over conventional approaches when trying to figure out how managing soil affects the tiny organisms in the soil (10–12).

First, DNA-SIP tells us exactly which organisms are active instead of guessing based on how many there are. Agricultural management practices alter soil microbial communities, with the magnitude of these effects varying according to the intensity of disturbance. Therefore, distinguishing between metabolically active and inactive organisms is crucial for understanding microbial community function (13). This point is illustrated by the authors’ observation that many carbon-cycling bacteria, while too rare to detect with standard sequencing methods, were successfully identified via isotopic incorporation.

Second, the temporal dimension of DNA-SIP provides insights into the dynamics of microbial responses that are impossible to obtain from just looking at a single moment or snapshot. The authors tracked how carbon was used over a month and found big timing differences in how bacteria reacted based on how the soil was treated. This temporal resolution is crucial for understanding how management practices influence the coordination of microbial activities (e.g., between tillage treatments).

Third, DNA-SIP can also tell the difference between when carbon is processed first and later (i.e., primary and secondary processing). By comparing when isotopes are used with peak mineralization activity, the authors figured out if bacteria were directly assimilating substrates or acquiring carbon through secondary processing of microbial products. This difference is key for interpreting the dual-labeling patterns observed in tilled soils.

**Fig 1:**
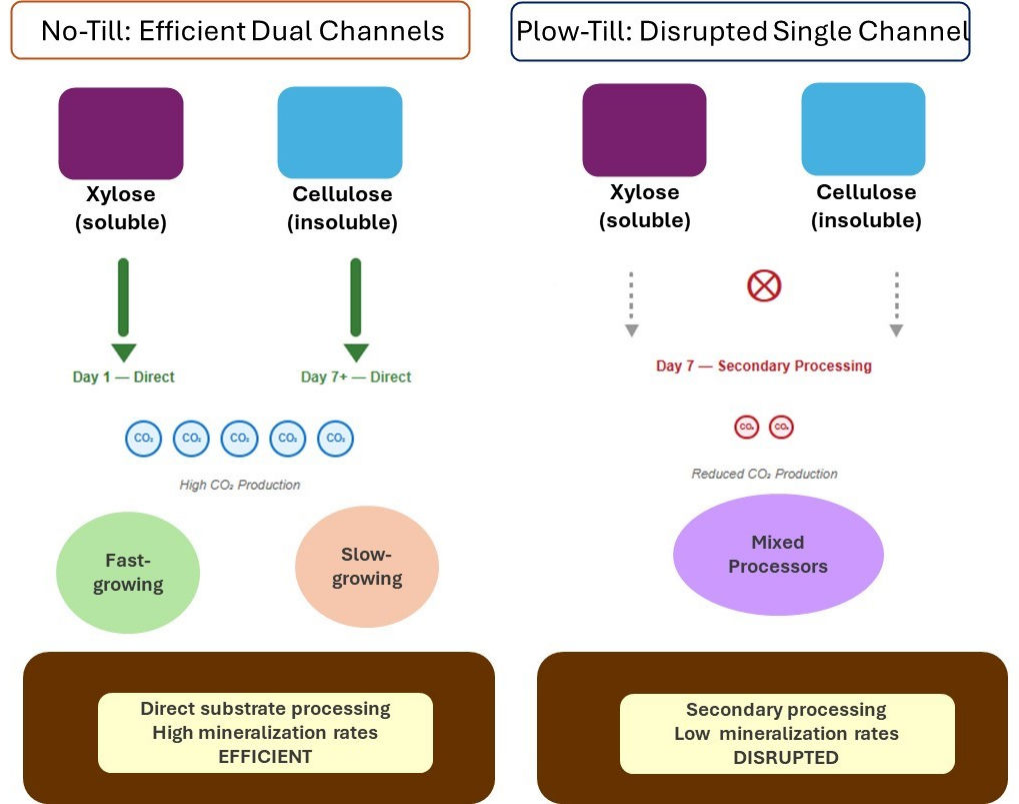
Tillage effects on bacterial carbon processing pathways after 42 years of management. No-till soils exhibit dual processing channels with direct substrate utilization: fast-growing bacteria process xylose on day 1, while slow-growing bacteria process cellulose from day 7+, resulting in efficient carbon mineralization (large CO₂ bubbles). Plow-till soils show disrupted processing where bacteria engage in secondary scavenging of both substrates around day 7, leading to reduced mineralization efficiency (small CO₂ bubbles). Thick solid arrows indicate direct processing; thin dashed arrows indicate secondary processing. Figure created using data from Schaedel et al.

The most striking finding of Schaedel et al. (6) is the identification of fundamentally different patterns of bacterial carbon use between tillage systems (Fig. 1). In no-till soils, bacteria break down xylose and cellulose using separate pathways, referred to by the authors as channels, with each channel having its own group of growth-adapted bacteria. High ribosomal RNA copy number bacteria rapidly process bioavailable xylose on day 1, while lower copy number organisms slowly break down cellulose over weeks. This temporal and functional separation reflects what ecologists call resource partitioning (14), where community members divide resource use among themselves to reduce competition and increase overall efficiency.

In contrast, tilled soils show a fundamentally different pattern where the same bacterial groups handle both xylose and cellulose, but only days after peak mineralization activity has occurred. Having dual-incorporator bacteria might seem advantageous because it represents broader metabolic capability. However, the authors demonstrate that this pattern reflects disrupted community organization rather than metabolic versatility. The fact that most dual labeling occurs on day 7—well after peak xylose mineralization on day 2—suggests these bacteria are scavenging carbon products from earlier microbial processing rather than directly metabolizing the original substrates. These findings challenge conventional assumptions about microbial community function and explain the mechanistic basis for observed differences in carbon cycling between management systems.

These patterns give us key insights into how tillage affects soil carbon cycling. Rather than simply altering community composition, tillage appears to fundamentally change how those bacterial groups work together. The authors propose that tillage disrupts the natural progression of soil bacteria, leading to fewer bacteria with specialized jobs and more bacteria that can do a bit of everything (i.e., generalists). This mechanistic understanding helps explain some confusing observations from previous studies. The often-modest correlations between community composition and ecosystem function in agricultural soils (15, 16) may reflect the difficulty of inferring functional organization from taxonomic data alone. Similarly, the context-dependent responses of soil carbon to conservation practices (17, 18) may be because different management practices affect functional community organization rather than just diversity or abundance.

DNA-SIP’s ability to track changes over time also gives clues about how stable management practices are. Bacterial responses in tilled soils have long delays, suggesting these communities might not handle environmental changes well. This could impact things like nutrient cycling and plant growth. From a practical point of view, these results give a base for improving farming practices to store more carbon in the soil. No-till management keeps specific bacterial groups that are better at processing organic matter, which explains why this method helps build up soil carbon. Instead of just reducing physical disruption, these methods seem to keep the soil community organized in a way that helps hold onto carbon.

The substrate-specific nature of management effects also has important implications for agricultural systems. Since xylose and cellulose respond differently to tilling, management success can depend on crop residue composition. Systems with higher proportions of readily decomposable compounds might show different responses to conservation practices than those dominated by recalcitrant materials. This can help create management plans for different crops, soils, or climates. When farmers understand how management practices change microbial communities, they can adjust them to get better land use and aid the environment. DNA-SIP’s success implies it can be used in many ways to understand agricultural systems. The approach could be further extended to evaluate other management practices, like cover cropping or fertilization, providing mechanistic insights into their effects on soil carbon cycling.

The temporal framework developed by Schaedel et al. also helps us understand the lasting effects of management. Extending isotopic labeling studies across seasons or years could reveal how bacterial carbon-processing networks develop and persist under different management regimes, providing insights into the stability and reversibility of management-induced changes.

Schaedel and colleagues have shown how DNA-SIP can change our understanding of agricultural soil systems. By directly tracking how bacteria take in carbon, they show functional patterns that conventional molecular approaches couldn't detect. Their finding that tilling really changes how bacteria process carbon gives us a better idea of how farming affects carbon moving through the soil. If we combine DNA-SIP with other multi-omics approaches, such as stable isotope-assisted metabolomics (19), we could get an even better sense of how soil systems work. As farming faces more pressure to be sustainable and productive, this kind of understanding will be key for making smart farming plans that make the most of both what nature gives us and what crops we grow.
